# What’s New in Gastric Cancer: The Therapeutic Implications of Molecular Classifications and Future Perspectives

**DOI:** 10.3390/ijms19092659

**Published:** 2018-09-07

**Authors:** Giuseppe Tirino, Luca Pompella, Angelica Petrillo, Maria Maddalena Laterza, Annalisa Pappalardo, Marianna Caterino, Michele Orditura, Fortunato Ciardiello, Gennaro Galizia, Ferdinando De Vita

**Affiliations:** 1 Division of Medical Oncology, Department of Precision Medicine, School of Medicine, University of Campania “Luigi Vanvitelli”, Via Pansini n.5, 80131 Naples, Italy; luca.pompella@icloud.com (L.P.); angelic.petrillo@gmail.com (A.P.); marilena_laterza@yahoo.it (M.M.L.); annalisa.pappalardo88@gmail.com (A.P.); caterinomarianna@gmail.com (M.C.); michele.orditura@unicampania.it (M.O.); fortunato.ciardiello@unicampania.it (F.C.); 2 Division of GI Tract Surgical Oncology, Department of Cardio-Thoracic and Respiratory Sciences, School of Medicine, University of Campania “Luigi Vanvitelli”, Via Pansini n.5, 80131 Naples, Italy; gennaro.galizia@unicampania.it

**Keywords:** gastric cancer, molecular classifications, targeted therapy, immunotherapy

## Abstract

Despite some remarkable innovations and the advent of novel molecular classifications the prognosis of patients with advanced gastric cancer (GC) remains overall poor and current clinical application of new advances is disappointing. During the last years only Trastuzumab and Ramucirumab have been approved and currently used as standard of care targeted therapies, but the systemic management of advanced disease did not radically change in contrast with the high number of molecular drivers identified. The Cancer Genome Atlas (TCGA) and Asian Cancer Research Group (ACRG) classifications paved the way, also for GC, to that more contemporary therapeutic approach called “precision medicine” even if tumor heterogeneity and a complex genetic landscape still represent a strong barrier. The identification of specific cancer subgroups is also making possible a better selection of patients that are most likely to respond to immunotherapy. This review aims to critically overview the available molecular classifications summarizing the main druggable molecular drivers and their possible therapeutic implications also taking advantage of new technologies and acquisitions.

## 1. Introduction

During the last years, “precision medicine” has deeply changed the therapeutic landscape of several malignancies. The customization of healthcare led to a global and significant improvement in cancer management and has faded the “one size fit-all” era. However, in contrast to the steady increase in survival observed for most cancer types, advances have been slow and difficult for gastric cancer (GC).

In fact, despite some remarkable innovations there is still an urgent need for a deeper understanding of the genetic and molecular background of this cancer and for novel treatment approaches.

Several therapeutic strategies have been already investigated and new molecular classifications have been proposed, nevertheless the prognosis of patients with advanced disease remains overall poor with a median overall survival (OS) of about 11 months and disappointing five-year survival rate of approximately 25–30% [1,2], even if a correct “continuum of care” is making longer survivals less anecdotical.

Only trastuzumab (anti-HER2 monoclonal antibody) and ramucirumab (anti-VEGFR monoclonal antibody) have proven to be successful weapons among all of the several molecular drivers identified, but currently still lacking of any clinical utility; for these reasons, the standard systemic chemotherapy still represents a “forced” mainstay of the treatment of advanced disease.

All of these efforts led to a fundamental acquisition: gastric cancer should be considered to be a collection of different molecular entities, rather than a single homogeneous disease.

As a matter of fact, in contrast with the overall declining trends for the four major malignancies, gastric cancer has still one of the highest estimated mortality rate, representing the third leading cause of cancer-related death (8.8%) with alarming statistics that make it one of the most lethal disease in the world (723,000 estimated deaths out of almost one-million new cases per year worldwide) [3].

Even if its incidence significantly fell over the last decades it still represents the fifth most common cancer (6.8% of all) with 10,800 new diagnoses still expected in 2018 only in the United States (US) [4].

These numbers require the biggest effort and attention on this cancer in order to investigate its heterogeneity and to maximize treatment outcomes.

The biggest challenge for the next future will be to understand which cancer subgroup deserves a specific targeted agent, also reconsidering drugs that have failed at an early research stage through the definition of more precise molecular drivers by using new technologies, such as patient-derived xenograft (PDX) and tumor organoids or an integrated genomic analysis, and consequently to minimize the amount of patients who receive a same treatment without any molecular selection.

This review aims to critically overview the available molecular classifications and the latest findings for GC and to outline the possible future scenarios and implications of these acquisitions.

## 2. Where It All Began: The “Old Dear” Histology

Gastric cancer of epithelial origin (GC) is today recognized as an extremely heterogeneous disease both in the clinical presentation modes as well as histological appearances and molecular basis. A first attempt to define GC heterogeneity was to look at it by microscope as performed by Lauren P. [5], who identified two main types of GC on histological bases: the first one called “intestinal”, as it displayed features characteristic of intestinal mucosa (and it was thought to arise from intestinal metaplasia of the stomach), and the other one called “diffuse” because the cancer cells, often poorly cohesive, diffusely infiltrated the gastric wall. The World Health Organization (WHO) classification of tumors of digestive system [6], on the other hand, classifies GCs, according to their histological appearance, in “tubular adenocarcinomas” (that contain dilatated and branching tubules, with cytological atipia varying from low to high-grade), “papillary adenocarcinomas” (typically well-differentiated exophytic carcinomas with elongated finger-like processes), “mucinous adenocarcinomas” (>50% of the tumor contains extracellular mucinous pool), and “signet-ring cell adenocarcinomas” (more than 50% of the tumor consists of isolated or small groups of malignant cells, highly infiltrative, containing intracytoplasmatic mucin), the latter one resembling those that are classified as “diffuse type” in the “Lauren classification”.

## 3. Two Steps Towards Precision Medicine: Molecular Classifications

This histological heterogeneity is only the tip of the iceberg if we think about the GC underlying molecular complexity, and, as a matter of fact, many molecular classifications have succeeded over the years (immunohistochemistry based, genomic based, proteomic based, etc.): in this section, we have tried to report the most significant, also in relation to novel therapeutic possibilities.

The first molecular attempt to classify comprehensively GC at molecular level was made by Patrick Tan’s group in Singapore [7]: they analyzed 60 GC samples (from 60 patients) by expression microarrays and comparative genomic hybridization and identified three molecular GC subgroups: “tumorigenic”, “reactive”, and “gastric-like”.

Each subtype was associated to a different biological function; for example, “reactive” group highly expresses endothelial growth factors and appears more sensitive to anti-angiogenic strategies. However, no associations were found between each subtype and Lauren’s histology or tumor grading, but, when survival analysis was performed, “gastric-like” tumor emerged as the most favorable subtype in a statistically significant way.

In 2011, the same Singapore group proposed another classification of GCs in G-INT (genomic intestinal) and G-DIF (genomic diffuse) [8]. Differently from others, the authors did not start their research characterizing primary human tissue, but they used a panel of 37 GC cell lines: in that way, they identified a “gene expression signature” of 171 genes that are able to distinguish these two intrinsic GC subtypes, the first one called “G-INT” because more related to Lauren’s intestinal subtype and the other one “G-DIF” more related to diffuse subtype. Moreover, this classification was also validated on a clinical cohort of 270 GC patients, with important prognostic informations: G-DIF tumors showed a statistically significant worse overall survival when compared to G-INT tumors, while Lauren’s histologies were not prognostic. Furthermore, predictive informations came out from in vitro experiments on 28 cell lines, with possibly relevant implications for patient’s care: G-INT cell lines were more sensitive to 5-fluorouralcil and oxaliplatin, while G-DIF resulted in being more sensitive to cisplatin. On that basis, authors designed a prospective “genomic-guided” chemotherapy trial (NCT01100801) [9], in which GC patients were allocated to “oxaliplatin arm” or “cisplatin arm” based on their intrinsic subtype; indeed, trial data were recently published [10], with some disappointing results since, although G-INT GC patients allocated to Oxaliplatin arm showed a deeper level of response, overall survival was better in G-INT patients that were allocated to cisplatin arm, leaving the authors a little bit confused, as well as the readers.

In the same years, Manish Shah proposed a new GC classification that is based on epidemiological and topographic tools [11], recognizing three subtypes: diffuse, proximal non-diffuse, and distal non-diffuse GC. Proximal non-diffuse GC arises in the cardia and it is preceded by precursor glandular dysplasia in the setting of chronic inflammation, but, differently from distal non-diffuse GC where inflammation is more related to H. Pylori infection, in proximal tumors, phlogosis is caused by gastric acid reflux [12]. To note, many tyrosine kinase receptors, like HER-2 (Human epitelial growth factor receptor 2) [13,14], EGFR (Epidermal growth factor receptor) [15], and c-MET (Mesenchymal epithelial transition factor receptor) [16] are more frequently expressed or amplified in proximal tumors when compared with distal tumors. Moreover, glycolysis and gluconeogenesis pathways are also upregulated in this subtype [10]. Distal non-diffuse gastric cancers arise instead between the gastric body and pylorus, often preceded by chronic inflammation with aspects of intestinal metaplasia, both consequences of chronic H. Pylori infection. These tumors frequently display an intestinal histological appearance (according to Lauren) and upregulate vascular endothelial growth factor and many other angiogenic pathways [17]. Finally, diffuse GC is characterized by diffuse pattern of infiltration: the complete loss of adherence properties by the cancer cells generate the so called “signet ring cells” histology, which is often due to CDH1 (Cadherin 1) tumor suppressor loss at genetic level. Other molecular aberrations in diffuse GC include FGF-R2 (Fibroblast growth factor receptor 2) tyrosine kinase receptor overexpression [18], PI3K (Phosphoinositide 3-kinase) signaling activity [19] and HER3 receptor overexpression [20]. Finally, expression of some Matrix Metallo-Proteases (MMP) is more frequent in diffuse versus intestinal cancers and it could contribute to tumor aggressiveness [21].

In 2013, Patrick Tan’s group again published a new attempt to molecularly classify GCs [22]: this time the authors performed a “consensus hierarchical clustering” of 248 GC samples and identified three major subtypes (“mesenchymal”, “proliferative”, and “metabolic”). The “mesenchymal” subgroup was so called because of the high activity of EMT (epithelial-to-mesenchymal transition) pathway: indeed, this subtype has very high levels of CDH2 (*N*-cadherin) transcripts and low levels of CDH1 (E-cadherin), typical of mesenchymal cells. However, many other pathways characterize this GC subtype:

(1) Cancer Stem Cells (CSCs) pathway: very high levels of CD44 (a marker of CSC) are described, with a more frequent poor differentiated histology (a surrogated marker of CSC).
(2) p53 pathway.
(3) Transforming Growth factor Beta (TGF-B) pathway.
(4) Vascular endothelial growth factor (VEGF) pathway.
(5) mTOR pathway (similarly to Shah’s “diffuse subtype”).
(6) Sonic Hedgehog (SHH) pathway: a very well defined pathway active in stem cells.

To note, mesenchymal subtype is more associated to Lauren’s diffuse dubtype than the other two (60% of tumors in this category are diffuse type), as well as the G-DIF (92%).

The “proliferative” subtype shows the high expression of a large set of genes correlated to cell cycle (*E2F*, *MYC*, *RAS*, etc.). These tumors are frequently correlated to Lauren’s intestinal type (74%) and G-INT subgroup (71%) with very high levels of p53 mutations and copy number amplifications (in CCNE1 locus, MYC locus, KRAS locus, to name a few). Lastly, the “metabolic” subtype highly expresses metabolic-related genes and digestive-related genes, the latter ones being typical also of normal gastric mucosa. Therefore the authors hypothesized that this subtype is closer to the normal gastric mucosa than the other two, even in terms of gene expression profile. An intermediate step between the normal mucosa and this cancer subgroup could be the SPEM (spasmolytic-polypeptide-expressing metaplasia), whose genes are highly expressed by the metabolic subtype.

Although these subtypes are molecularly very distinct, no differences in terms of survival were identified. However, metabolic subtype seems to be more sensitive to 5-fluorouracil than the other two, perhaps in relation to low levels of thymidylate synthetase, while the mesenchymal subtype (probably due to “oncogenic addiction” to PI3K-AKT-mTOR pathway, as we discuss above) seems to be sensitive to several drugs that block PI3K or mTOR, opening the way for a more precise therapy for GC.

In 2014 Leung et al. [23] demonstrated the multidimensional genomic landscape and the molecular complexity of gastric adenocarcinomas performing an integrative genomic analysis on a dataset of 100 diffuse and intestinal GC samples (tumor and normal tissue paired). Using four different platforms (whole-genome sequencing, DNA copy number, gene expression, and methylation profiling), they identified the main aberrant pathways and subtype-specific genetic and epigenetic perturbations. In addition to the known mutated driver genes (TP53 frequent in both subtypes, ARID1A in Epstain Barr Virus-related (EBV) or microsatellite instability-related cancers and CDH1 in diffuse-type), authors have been able to describe new highly recurrent significant mutations (i.e., MUC6, CTNNA2, GLI3, RNF4) and particularly the high prevalence of *RHOA* (Ras homolog gene family, member A) mutations in diffuse-type tumors (14.3% vs. 0% in the intestinal-type, *p* < 0.001). These mutations determines defective RHOA pathway leading to aberrations in the adhesion functionality and escape from programmed cell death that occours when anchorage-dependent normal cells detach from the extracellular matrix, also demonstrating the possible tumor suppressive role of RHOA in this subtype. The study was one of the first to prove the deep molecular differences and to highlight the specific genetic perturbations covered below different histological features.

Few months later, the Cancer Genome Atlas (TCGA) investigators published the most important and comprehensive study that we have to date on molecular GC classification [24].

The authors characterized 295 GC tumor samples using six different molecular platforms (copy number alterations, whole exoms sequencing, mRNA sequencing, miRNA sequencing, DNA methylation analysis, and phosphoproteomic analysis) and identified four molecular subtypes: EBV-related, MSI-H (Microsatellite instability-high), Genomically Stable (GS), and Chromosomal Instability (CIN). The first one (9% of cases) is called “EBV-related”, because it is characterized by Epstein Barr virus infection in the cancer cells: these tumors are mainly located in the gastric fundus or body and show extensive DNA promoter hypermethylation (a marker of “gene silencing”). Moreover, they have the highest frequency of PIK3CA (encoding for the catalytic alfa subunit of PI3K kinase) mutations (80%), as well as amplifications of *JAK2* (Janus kinase 2) or *PD-L1/L2* genes, making this subtype “ideally” the most sensitive to PI3K or PD1/PDL1 inhibition (as we will see later). The second group (22% of cases) was called “MSI” because it was characterized by genomic instability, due to a deficient DNA mismatch repair system, and lacked targetable amplifications. This subtype shows hypermethylation of MLH1 promoter region (leading to MLH1 silencing)—the cause of MSI status—and a very high mutation rate with hotspot mutations involving several genes like *HER2* (5%), *EGF-R* (5%), *HER3* (14%), *JAK2* (11%), *FGFR2* (2%), *MET* (3%), and *PIK3CA* (42%). To note, the BRAF^V600E^ mutation commonly seen in MSI-H colorectal cancer was universally absent. Finally, gastric MSI tumors have a very high rate of PD-L1 expression that, when associated with the high number of mutation-associated neoantigens, could make them very sensitive to checkpoint inhibitors.

The third group is called “Genomically Stable” (GC) and it accounts for 20% of TCGA dataset: it lacked somatic copy number aberrations and was more related to Lauren’s diffuse histology than the other ones. A pathway frequently destroyed in this subtype is that related to “cell adhesion”, with the most relevant genes mutated CDH1 (26%), RHOA (15%), and chromosomal translocation involving CLDN18 and ARHGAP (15%). The last group is characterized by chromosomal instability (50% of cases), and it thus called “CIN”. Gene amplifications are very frequent, with involvement of different tyrosine kinase receptors or related pathways: HER2 (24%), EGF-R (10%), HER3 (8%), JAK2 (5%), FGFR2 (8%), MET (8%), PIK3CA (10%), and KRAS/NRAS (18%).

After all, if we move to clinical significance of this classification, not reported in the original paper, Sohn et al. [25], while using gene expression data from one of the TCGA cohort (*n* = 262), developed a robust subtype-based prediction model with the EBV subtype resulting as the one associated with the best prognosis and the “GS” subtype with the worst. Moreover, MSI and CIN subtypes had an intermediate prognosis, with a poorer overall survival than those with EBV+ but better than those with GS subtype. The authors also found important predictive informations, as the CIN subgroup experienced the biggest benefit from adjuvant chemotherapy, while the GS subtype the smallest.

One year later, the Asian Cancer research group (ACRG), analyzing 300 gastric tumor samples by two molecular platforms, provided a new GC classification, and identified four subtype [26]: “MSI”, “MSS/EMT”, “MSS/p53+” (p53 active), and “MSS/p53−” (p53 inactive). One of the strengths of ACRG classification is to precisely correlate each molecular subtype with clinical information, like prognosis, recurrence frequency (after surgery), and pattern of recurrence (i.e., peritoneal versus hepatic).

The MSI subtype (23% of cases), as in the TCGA cohort, was found to be hypermutated due to the frequent loss of MLH1. These tumors occur mainly in gastric antrum (75%), they are preferentially of intestinal subtype (>60%) and >50% of subjects are diagnosed at an early stage (I/II). Genes frequently affected by mutations are KRAS (23%), ALK (16%), ARID1A (44%), and those related to PI3K pathway (42%). To note, this group is associated with the best overall prognosis and the lowest frequency of recurrence (22%) of the four subtypes. The MSS/EMT (epithelial to mesenchymal transition) group (15% of cases) occurs at significantly younger age and shows mainly diffuse histology. It is characterized by a very low mutation rate when compared with other MSS groups (*p* < 0.001), but with the frequent loss of CDH1 expression, especially in a large set of signet ring cells adenocarcinomas. More importantly, the majority of subjects (>80%) in this subtype are diagnosed at stage III/IV: therefore the MSS/EMT has the worst prognosis, with the highest chance of recurrence (63%), mainly at the peritoneal site.

MSS/p53+ tumors (26% of cases) show frequent EBV positivity (with some overlap with the “EBV-related” subtype of TCGA) and a preserved activity of p53 tumor suppressor gene. Frequent mutations hit APC, ARID1A, KRAS, PIK3CA, and SMAD4. This subtype is also associated with the best overall prognosis after MSI subtype.

MSS/p53− tumors (36% of cases) present overlap with the CIN group from TCGA, showing the highest prevalence of p53 mutations and recurrent focal amplifications of tyrosine kinase receptors, like ERBB2 or cell-cycle modulators, like CCNE1 or CCND1.

## 4. Comparison of TCGA and ACRG Data

The TCGA and ACRG classifications are partially overlapping and complementary models: they both identified an MSI group of tumors characterized by high mutation frequency and best prognosis. While CIN and GS subtypes are present across all the ACRG groups, it is noteworthy that TCGA EBV+, GS, and CIN subtypes are enriched in ACRG MSS/p53+, MSS/EMT, and MSS/p53− respectively.

However, while CDH1 and RHOA mutations are highly prevalent in the TCGA GS subtype, in ACRG MSS/EMT, these mutations are extremely rare, making these two subtypes absolutely not equivalent or synonyms.

Possible reasons for this partial overlap between these classifications include differences related to the patient population (Korea in ACRG versus USA and Western Europe in TCGA), tumor sampling (mainly diffuse in ACRG), and technological platforms (six different molecular platform in TCGA (exome sequencing, copy number analysis, mRNA-miRNA-methylation analysis), versus only mRNA expression and targeted gene sequencing in ACRG).

Although their limitations these two classifications represent today the most important groundwork for the development of targeted therapies inspired to a concept of “biologically-guided tumor treatment”, as well as patients stratification for clinical trials and improved prognostication.

## 5. Clinical Implications of Molecular Classifications

How can we use this enormous amount of information for a clinical application? Unfortunately, neither the TCGA nor AACR classifications can be currently used for patients’ stratification and selection, as for many of the identified mutated genes the functional relevance of mutation is not known and, more importantly, they are not yet druggable. 

In this section, we individually consider the most relevant molecular targets that are identified in gastric cancer and we discuss their potential therapeutic implications (Table 1).

### 5.1. HER2

HER2 (Human Epidermal Growth Factor Receptor II) or ERBB2 (Avian erythroblastosis oncogene B), encoded at chromosome 17q21, is a well-defined tyrosine kinase receptor often acting as proto-oncogene in many human cancers. Oncogenic mechanisms that hit HER2 are represented by gene amplification (determining protein over-expression) or less commonly activating mutations.

HER2 lacks of a known exogenous ligand and it is transactivated by the interaction with other HER family members (EGFR or HER3 overall) or other tyrosine kinase receptors. Its activation leads to a complex cascade of transduction events within the cytoplasm, that converge on two fundamental signaling pathways: the RAS-MAP (mitogen activated protein) kinase pathway and the PI3K-AKT pathway, both determining cell survival, proliferation and migration.

In GC HER2 overexpression is mainly due to gene amplification: it occurs more frequently in proximal tumors (more than 30% of cases), than in distal cancers (less than 20%), mainly arising from the gastric body. Furthermore, Lauren intestinal subtype shows a higher expression of HER2 (up to 34%) than diffuse subtype (6%), while, concerning to TCGA classification, CIN tumors more often express HER2 as consequence of gene amplification (as mentioned above).

Different strategies to target HER2 were developed over the years: monoclonal antibodies (like trastuzumab) that bind to the extracellular domain of the receptor, determining receptor down-regulation or antibody-dependent-cytotoxicity (ADCC), and TKIs (tyrosine kinase inhibitors), that inhibit signaling cascade through the blockade of receptor kinase activity.

The pivotal phase III ToGA (Trastuzumab for Gastric cancer) trial [27] showed that, in HER2 positive GCs, the addition of trastuzumab to standard platinum-based first line treatment was effective, with a median overall survival (mOS) of about 13.8 months in the experimental arm versus 11.1 in the standard one (HR: 0.74; *p* = 0.0046). This OS still represents the highest ever reached in a phase III trial recruiting GC patients. The greatest benefit was observed in high HER2 expressing patients (IHC3+ or IHC2+/FISH+), with a mOS of 16 months versus 11.8 in low HER2 expressing patients (IHC0-1+/FISH+). Therefore, this trial led to the approval of trastuzumab in HER2 positive GC, in the first line setting for patients with IHC3+ or IHC2+/FISH+.

Based on the extraordinary results of the Cleopatra Trial in HER2 positive breast cancer [28], it has been speculated that also in GC the addition of pertuzumab (another monoclonal antibody targeting a different HER2 domain than trastuzumab) to trastuzumab itself and platinum-based chemotherapy could improve the ToGA survival rates, leading to JACOB Trial design. Unfortunately, this study was almost negative [29] because the mOS was 17.5 months in experimental arm versus 14.2 in the standard (HR: 0.84; *p* = 0.0565), a difference that did not find statistical significance.

Trastuzumab-emtansine (TDM-1) is an antibody-drug conjugate that is widely used in second line setting for metastatic HER2 positive breast cancer [30]. This drug was also studied in second line therapy of HER2 positive GC (previously treated with trastuzumab) within the GATSBY phase III trial [31]: unfortunately, TDM-1 therapy was not superior to standard taxanes (mOS 7.9 months versus 8.6 respectively, HR: 1.15, *p* = 0.86), although with lower incidence of adverse events.

Another strategy to target HER2 consists of the inhibition of kinase activity with small molecules TKIs, like Lapatinib (a multi-kinase inhibitor with a strong activity against EGFR and HER2). This molecule was tested in two randomized phase III trials enrolling GC patients with advanced disease: the LOGIC and TYTAN trials. 

The LOGIC trial [32] tested lapatinib in combination with capecitabine plus oxaliplatin versus chemotherapy alone in the first line setting of HER2 positive GC patient. The trial results were negative, because mOS (the study primary endpoint) was 12.2 months in experimental arm versus 10.5 in the standard (HR: 0.91, *p* = 0.349), a statistically not significant difference, although secondary endpoints like ORR or PFS were in favor of Lapatinib arm.

Furthermore, lapatinib has been tested in second line setting within the TYTAN phase III trial [33], in wich 261 previously treated Asian GC patients were enrolled to receive lapatinib plus paclitaxel or paclitaxel alone (to note only 15 patients [6%] had previously received trastuzumab in first line). Once again, the results were negative, with insignificant differences between the two arms for OS and PFS.

Due to the disappointing results of these trials (JACOB, GATSBY, TYTAN, LOGIC), many researchers began to study mechanisms of targeted therapy resistance in GC, when considering that also patients who achieved a significant response to first line trastuzumab-based treatment can develop resistance within a few months [34].

In effect, one main bias of the second-line trials, especially the GATSBY trial, seems to be the absence of tumor rebiopsy (for example at metastatic site) at screening, taking for granted that the tumor was still HER2 positive on the basis of the basal diagnostic biopsy.

An Italian study [35] clearly showed that the acquired resistance mechanism to trastuzumab-based first line treatment could be the loss of HER2 receptor, especially for patients with dubious immunohistochemistry (IHC2+/FISH+), speculating that HER2 negative clones are positively selected by the first line anti-HER2 therapy and could subsequently expand. In that way, the negative results of the GATSBY study could be related to the fact that, in a significant proportion of cases, they have treated with TDM-1 patients who were *de facto* HER2 negative at the beginning of the second line.

Another possible mechanism of acquired resistance to trastuzumab is likely due to co-existing molecular alterations within the HER2 positive tumor clones, as clearly showed by Pietrantonio et al. [36]: mutations of EGFR/MET/KRAS/PIK3CA/PTEN or the amplifications of EGFR/MET/KRAS can co-occur in HER2 positive cells and could explain the lack of trastuzumab efficacy and/or the appearance of resistance.

In any case, to date, no standard anti-HER2 treatment is available in trastuzumab refractory HER2+ patients, and standard chemotherapy with taxanes (mainly the combination of Paclitaxel plus ramucirumab) or irinotecan is recommended.

### 5.2. EGFR

Epidermal Growth Factor Receptor (EGFR) or ERBB1 is a transmembrane tyrosine kinase receptor, expressed approximately in 30% of GC [37], especially those with chromosomal instability (“CIN” subtype of TCGA). This molecule represents the second most important receptor (after HER2) in GC pathogenesis: its overexpression is associated with poorly differentiated histology, vascular invasion, and potentially shorter survival [38].

Several studies evaluated the safety and efficacy of different anti-EGFR drugs, on the basis of preclinical works [39]: anti-EGFR therapies include—as we just discussed for HER2—monoclonal antibodies (like cetuximab or panitumumab) and TKIs (gefitinib, erlotinib).

Initial phase II trials combining these agents with cytotoxic chemotherapy in unselected patient population identified high response rates for the first line setting (from 41 to 65%) [40,41]. Unfortunately, all of the phase III trials investigating the role of anti-EGFR therapy in GC were negative.

The EXPAND study [42] randomized GC patients in first line setting between cetuximab plus capecitabine-cisplatin versus chemotherapy alone, showing no advantage for cetuximab arm (mPFS 4.4 months versus 5.6 months, *p* = 0.32). The patient recruitment was unselected for EGFR positivity, although in a post-hoc analysis the highest survival benefit was observed in a small subset of patients with high EGFR expression (representing probably EGFR amplified tumors).

The REAL-3 trial [43] demonstrates that adding panitumumab to epirubicin-oxaliplatin-capecitabine was even detrimental, as the mOS for the experimental arm was 8.8 months versus 11.3 months for the standard one (HR: 1.37, *p* = 0.013).

The deep failure of anti-EGFR drugs in gastric cancer can be explained mainly with the lack of a proper patient selection. In fact, a recent work by Catenacci et al. [44] showed that EGF-R amplified tumors (5% in Chicago casuistry), some of which were treated with anti-EGFR drugs, seems very prone to respond to cetuximab or ABT-806 (an investigational anti-EGFR drug), with an ORR of 58%, a DCR of 100%, and a mPFS of about 10 months. Thanks to next generation sequencing (NGS) and circulating tumor DNA (ctDNA) studies, the authors also showed the mechanisms of resistance to anti-EGFR drugs, such as the presence of EGF-R negative tumor clones, KRAS mutation/amplifications, PTEN deletion, and NRAS/HER2/MYC amplifications. 

This study definitively demonstrates that EGF-R amplification is able to predict response to anti-EGFR therapies, despite the negative results in prior unselected phase III trials (EXPAND and REAL-III), but also that mechanisms of resistance exist and could be detected by novel technologies, like NGS and ctDNA.

### 5.3. C-MET

*MET* (Mesenchymal-Epithelial Transition) oncogene, also called Hepatocyte Growth Factor Receptor (HGF), is a receptor tyrosine kinase that appears to be deregulated in many human cancers [45], such as breast, colorectal, lung, pancreatic, hepatic and—not least—gastric cancer.

Its activation requires binding to HGF (the soluble ligand of MET), the so-called “canonical activation”, but can also occur without HGF, through a cross-talk with other receptors (the “non canonical” pathway) [46]. MET signaling in GC is related to worse prognosis [47], because HGF/MET activity is involved in cancer growth, invasion, angiogenesis, and epithelial-to-mesenchymal transition.

The main known mechanism of MET overexpression in GC is gene amplification, which occurs in about 6% of the TCGA dataset (especially in CIN tumors). However, even tumors without gene amplification can express (or overexpress) MET, although it is not clear whether these tumors really depend on MET for survival and malignant properties.

Two monoclonal antibodies, Rilotumumab (an anti-HGF antibody) and Onartuzumab (an anti-MET antibody) were tested in clinical trials in GC: early reports [48,49] suggested that MET expression could serve as a predictive biomarker for anti-MET directed therapies, but in both phase III clinical trials evaluating Onartuzumab and Rilotumumab, the results were negative.

The METGastric phase III trial [50] evaluated the addition of onartuzumab to a chemotherapy backbone (mFOLFOX6), and enrolled 562 GC patients with HER2 negative/MET positive tumors (defined as score 1+, 2+, and 3+ by immunohistochemistry). The enrollment was stopped early due to sponsor decision, for a lack of efficacy in a phase II trial also assessing contemporary the role of onartuzumab in MET positive GC [51]. Unluckily, the addition of onartuzumab to mFOLFOX6 did not result in an improvement of OS (11 months in the experimental arm versus 11.3 in standard, HR: 0.82, *p* = 0.24) and PFS (6.7 versus 6.8 months, respectively, HR: 0.90, *p* = 0.43).

Negative results were obtained also with rilotumumab within the RILOMET-1 phase III trial [52], which used a different chemotherapy backbone (Epirubicin plus Cisplatin and Capecitabine). In that case, not only results were clearly negative with a detrimental effect (mOS was 8.8 in experimental arm versus 10.7 in the placebo group, HR: 1.34, *p* = 0.003), but study treatment was also stopped early, because an independent data monitoring found a higher number of deaths in the rilotumumab group than the placebo group.

There is a great discordance between the phase II rilotumumab study [48] and the RILOMET-1 results, because the MET+ patients treated with rilotumumab within the phase II (*n* = 41) had a mOS of 10.6 months when compared with 5.7 months of patients receiving placebo. Noteworthy, despite the cutoff to define MET positivity with immunohistochemistry was the same between phase II and III (expression ≥1+ in ≥25% of tumor cells), there was a relevant difference in the number of patients screened who were considered MET positive in phase II (64%) and phase III (81%) trial, for not known reasons.

Probably the main limit of RILOMET and METGastric trials is to have included mostly patients in whom MET was not a clear “driver” of the disease, since the highest expressing tumors (MET gene amplification) are under-represented, which can explain the negative results.

### 5.4. FGF

FGFR2 belongs to FGFR receptor family, which includes four different receptors and almost 23 different ligands, making it an extraordinary complex system [53]. The ligand-receptor interaction leads to initiation of a signaling cascade that lead, as for the majority of RTKs, to MAPK and PI3K-AKT pathways activation.

In GC, the first evidence of FGFR2 amplification has been described in 1990 [54], analyzing KATO III cell line. Moreover in the TCGA dataset almost 9% of patients within the CIN subtype presented FGFR2 gene amplification, which convinced researchers to test the FGFR inhibitors in FGFR2 amplified GC.

One molecule tested is AZD4547, a selective FGFR1,2,3 TKI with powerful preclinical activity in FGFR2 amplified GCs [55]. This drug was evaluated in the SHINE trial [56], in which GC patients displaying FGFR2 amplification or polysomy were randomized in second line setting between AZD4547 or paclitaxel. Trial results were negative, with a median PFS of 1.8 months in experimental arm versus 3.5 in the chemotherapy one. These very disappointing results have been mainly due to great intratumor heterogeneity for FGFR2 amplification—the FGFR2 status evaluated on archival tumor tissue may not reflect the molecular status of metastatic tumor at study screening—and to poor concordance between gene amplification and receptor expression. Therefore, an alternative predictive biomarker testing for FGFR2 is urgently needed, when considering that FGFR2-amplified tumors with FGFR2 overexpression, although at very low prevalence, exist and should be appropriately treated.

### 5.5. CLAUDIN18.2

Claudins are a well-known family of proteins that shape the fundamental part of tight cell junctions [57]. Within the normal gastric mucosa the isoform 2 of the Claudin18 (claudin 18.2 or CLDN18.2) is highly expressed, especially in differentiated epithelial cells, while it is quite absent in the gastric stem cell zone.

Claudin18.2 is retained in malignant transformation and is expressed in a significant proportion in primary tumors and their metastasis [58]. This molecule seems to be a good target especially in TCGA “GS” tumors that in a significant proportion (15% of cases) show a chromosomal translocation involving CLDN18 and ARHGAP.

In a phase IIb study (the FAST trial) [59], the role of Claudiximab (a chimeric monoclonal antibody against CLDN18.2 also known as Zolbetuximab-IMAB362) has been evaluated in combination with chemotherapy (epirubicin, oxaliplatin, and capecitabine [EOX]) versus chemotherapy alone in the first line setting (*n* = 161). This trial met its primary endpoint, because claudiximab significantly improved mPFS (7.9 months versus 4.8, HR: 0.47, *p* = 0.0001) and mOS (13.3 months versus 8.4, HR: 0.51, *p* < 0.001) as compared to EOX alone. A more pronounced benefit was observed in patients with very high CLDN18.2 expression (≥2+ intensity in ≥70% of tumor cells), making this combination a very appealing strategy for HER2 negative GC.

A notable point in the FAST study is that the outcomes in the EOX only arm were not similar to the corresponding landmark trial REAL 2 study (OS of 11.2 (REAL 2) vs. 8.7 (FAST), which could be due to patient selection.

Other trials are under development for CLDN18.2 positive gastric cancers such as the phase II trial with Zolbetuximab (NCT03505320—“ILUSTRO” trial) and a phase III trial with claudiximab is scheduled and expected.

Overall, the major limitation seems be the availability of the testing for CLDN18.2 and the finding of the ideal cut-off point for the CLDN18.2 levels, with the suggestion of studies comparing outcomes between low CLDN18.2 levels versus higher levels. The anti-claudin research is one of the best examples of how targeted therapy is clearly the future also of gastric cancer treatments.

### 5.6. VEGF/VEGFR

In the TCGA “CIN” subtype, vascular endothelial growth factor (VEGF), a crucial mediator of normal and pathogenic angiogenesis, is frequently amplified up to 7% of cases. Although initial studies with bevacizumab (a monoclonal antibody targeting VEGF-A) were negative, such as the AVAGAST trial [60] and the Asiatic AVATAR trial [61], in which bevacizumab was combined with platinum-based chemotherapy in first line setting, other antiangiogenic strategies continued to be investigated.

Ramucirumab, a fully human monoclonal antibody directed against VEGFR2 (Vascular endothelial growth factor receptor 2), which is the main receptor of the VEGF system implicated in oncogenic angiogenesis, has been used in the second line setting alone [62] or in combination with weekly paclitaxel [63].

Both studies were positive, with the REGARD trial showing a significant improvement in OS with ramucirumab alone versus BSC (mOS 5.2 months versus 3.8, respectively, HR: 0.776, *p* = 0.047) and the RAINBOW trial showing a significant superiority of combination arm (ramucirumab plus paclitaxel) versus paclitaxel alone (mOS 9.63 months versus 7.36 months, respectively, HR: 0.807, *p* = 0.017).

On that positive basis, ramucirumab has been tested in first line setting in combination with cisplatin-based standard chemotherapy within the RAINFALL trial [64]: although the study formally met its primary endpoint, with an improvement in mPFS from 5.4 months (placebo arm) to 5.7 months (ramucirumab arm) (HR: 0.75, *p* = 0.011), there was no survival benefit for patients treated with RAM + Capecitabin/Cisplatin versus placebo + Capecitabin/Cisplatin (mOS 11.2 months versus 10.7 months, HR: 0.96, *p* = 0.68), making the results negative *de facto* and not significant for clinical practice.

Therefore, the role of antiangiogenic agents seems to be essential in second line setting, but in the first line, like the AVAGAST and AVATAR trial, showed for bevacizumab, probably we need to better understand who are the patients that really benefit from this strategy.

### 5.7. PI3K Pathway

The PI3K/AKT/mTOR pathway is a fundamental promoter of cell growth, metabolism, survival, and cell migration: it is mainly activated by cell surface tyrosine kinase receptors, but in human cancer, many component of this pathway could be affected by activating mutation (PIK3CA) or inactivating genetic events (PTEN), like gene deletion.

Approximately, 80% of PIK3CA mutations occur at three recurrent hotspots: E545K and E542K in the exon 9, and H1047R in the exon 20 [65].

In gastric adenocarcinomas, PIK3CA is one of the most frequent mutated genes especially in EBV-related GC (almost 80% of cases are mutated) and “MSI” subtype (42% of cases), as shown by TCGA, making this molecule an appealing target to pharmacological inhibition.

One of the first study evaluating the block of PI3K-AKT-mTOR pathway in GC is the GRANITE-1 Trial [66], a phase III study in which 656 GC patients who progressed after previous one or two lines of systemic chemotherapy were randomized between everolimus (a mTOR inhibitor) or placebo. Results were almost negative, because the mOS (primary endpoint of the study) resulted 5.4 months with everolimus versus 4.3 with placebo (HR: 0.90, *p* = 0.124) and mPFS was 1.7 months versus 1.4, respectively (HR: 0.66, *p* < 0.001). The modest (and statistically significant) improvement in PFS suggested a potential benefit in selected patients: for example, PIK3CA/PTEN mutations could be predictive of mTOR inhibition, as suggested by Meric-Bernstam et al. [67].

Other studies with drugs that inhibit directly PI3K or AKT are in early phase clinical trials and to date no data are available.

## 6. Gastric Cancer in the Immunotherapy Era: A Hope for the Future

Immunotherapy deeply changed the therapeutic landscape for several malignancies (advanced melanoma, lung, urothelial, kidney cancer, etc.) determining a global outcome improvement completely unexpected until a few years ago by boosting the body’s natural defenses to fight cancer [68]. Gastric cancer is still late when compared to these others cancer types, even if some relevant results have been lately scored leading to a more confident vision for the future [69].

As already reported comprehensive molecular characterization performed by the TGCA group showed a relatively high mutational load (up to 10–15 mutations per megabase) in about 34% of gastric adenocarcinomas analyzed and a subset of tumors with microsatellite instability-high (MSI-H, 22%) or with an ideally favorable immune-environment (the “EBV-related” subgroup that shows molecular hallmarks of sensitivity to immunotherapy, such as intra- or peritumoral immune cell infiltration and PD-L-1/PD-L-2 expression), suggesting that also gastric cancer could be a promising “fertile soil” for immunotherapy, especially based on immune checkpoint inhibitors.

These checkpoints play critical roles for physiological homeostasis and for balanced immune responses and they are heavily involved in the immune escape mechanisms of GC as well.

Also, other previous preclinical evidence supports the idea that immunotherapy can be a successful anti-GC strategy, particularly regarding the T cell-based treatment protocols: cytotoxic T lymphocytes (CTL) and tumor infiltrating lymphocytes (TIL) [70,71].

Induced CTL cell culture technology (using specific peptides) led to the clinical tests based on adoptive transfer of CTLs in patients. The induced CTLs showed specific activity against tumor cells in vitro and against primary cell culture isolated from GC patients, suggesting that this strategy could be a kind of “vaccine” and adoptive immunogenic therapy as already preliminarily demonstrated in melanoma patients (up to 40% of antitumor immune responses in phase 2 trial) [72]. As a matter of fact CTLs from GC patients are able to identify specific tumor-associated antigens and to attack the autologous neoplastic cells triggering the immune responses against gastric cancer [73].

Furthermore, Kim et al. [74] also demonstrated the anti-gastric cancer activity of cytokine-induced killer cells (CIK) that are mainly T CD80+ cells isolated from human peripheral blood mononuclear cells cultured with IL-2 and anti-CD3 antibody. The CIK cells were capable of destroying human gastric cancer cells in vitro and to inhibit tumor growth in mouse model, indicating a potential role of CIK cells as adoptive immunotherapy for GC as well.

The transfer therapy with TILs requires first of all the T cell isolation from neoplastic tissue, then the in vitro expansion and finally the selection of tumor-specific T cells. The application of these protocols in GC patients is more difficult compared to melanoma because of an hardest surgical availability of adequate tumor tissue (only 30–40% of biopsies usually acceptable for the procedure) but this strategy seems particularly promising, as shown by the positive correlation between the presence of TILs and survival in ovarian, colorectal and pancreatic cancer [75,76,77] and it should be still encouraged and enforced. It is important to underline that in some cases TILs can even promote cancer development depending on the functional features of lymphocytic infiltrate [78]. These techniques and others (such as dendritic cell-based vaccination [79]) need to be deepened and optimized also for gastric cancer and they appear as a complex universe yet to be entirely discovered.

With regards to checkpoint inhibitors, we can find the strongest evidence that is currently available to support the approval for use of immunotherapy in GC with first promising results.

The KEYNOTE-012 trial [80] demonstrated in an early-phase the potential application of anti-PD1 therapy with pembrolizumab (humanized IgG4-k monoclonal anybody selective to bind PD1, currently approved by FDA in the US) in 39 patients that were affected by PD-L1 positive refractory advanced gastric cancer with promising overall response (22%, 95% CI 10–39). In the single arm phase 2 trial KEYNOTE-059 [81] this activity has been confirmed in an unselected population of patients with metastatic GC (cohort 1, *n* = 259) previously treated with two or more systemic lines of therapy. Objective response rate (ORR) was 11.6% but it reached 15.5% in patients who were PD-L1 positive (57.1% of all, cut-off of PDL-1 ≥1%) versus 6.4% in PD-L1 negative. The outcomes were significantly better when treatment were used in an early setting (third line ORR = 16.4% versus 6.4% in the fourth line), supporting the rationale to use immunotherapy as soon as possible in the natural history of the disease when the immune response is not compromised, and when population has been stratified according to MSI status (ORR = 57.1% in MSI-H; ORR = 9.0% in MSS), although only seven patients (4%) resulted with a microsatellite instability-high status. With a median OS of 5.6 months (secondary endpoint) a promising survival rate of 23.4% at 12 months has been described.

These results suggest the need for an accurate selection of patients in order to maximize the outcome of immunotherapy and to identify the subset of patients who respond favorably.

For this purpose, Kim et al. [82] designed an open label phase 2 trial with integrated genomic analysis of all baseline tumor tissue samples and genomic profiling of circulating tumor DNA (ctDNA) in order to classify the disease characteristics of responders and non-responders to immunotherapy. 61 Asian patients with metastatic or recurrent gastric cancer refractory to standard chemotherapy were enrolled and treated with Pembrolizumab, the population included six EBV-positive diseases and seven MSI-H. An ORR of 24.6% was observed but EBV-positive and MSI-H patients obtained dramatic responses to pembrolizumab (ORR, respectively, of 85.7% and 100%), furthermore activity was significantly higher in PDL-1 positive cancer (cut-off ≥ 1) when compared to PDL-1 negative (ORR of 50.0% versus 0.0%, *p* ≤ 0.001). In addition, other cancer subtypes such as genome stable, CIN and mesenchymal (defined by positive EMT signature) demonstrated poor responses to pembrolizumab, and particularly, the EMT subtype has been demonstrated to be a negative predictor of response to immunotherapy determining a poor survival.

Investigators additionally demonstrated a strong correlation between ctDNA mutational load and tumor mutational burden (TMB) and that decreasing ctDNA levels during the treatment are a powerful predictor of prolonged PFS and good response, opening the way to the use of ctDNA to identify patients that are likely to respond to pembrolizumab.

It is important also to outline that Pembrolizumab did not improve survival as second line treatment for PDL-1 positive advanced GCs according to findings from phase 3 KEYNOTE-061 trial recently published [83], even if the approved FDA indication for patients who have received at least two previous lines of treatment remains unchanged at current time. These negative findings added interpretative complexity to the knowledge about immunotherapy in GC.

Another anti-PD-1 drug that was successfully tested in the treatment of GC has been Nivolumab (fully human IgG4 monoclonal antibody). In the large randomized Asian phase 3 trial ATTRACTION-2 [84] Nivolumab showed a significant survival benefit in heavily pretreated patients (≥2 previous lines of treatment) with advanced gastric or gastro-esophageal junction cancer reaching an overall survival of 5.26 months as compared to 4.14 months in the placebo group (HR: 0.63–95% CI 0.51–0.78; *p* < 0.0001) and with an ORR of 11.2% (0% in the placebo arm). Interestingly remarkable survival rates were described at 12 months (26.2%) and at 18 months (16.2%) in the immunotherapy group, when considering the advanced setting of treatment. We could infer that there is a definite subset of patients that strongly benefits from the treatment and has durable responses.

A potential limit of this trial could be considered the lacking of molecular analysis and patients selection (the cohort was unselected according to PDL-1 status and tumor tissue samples was not mandatory at the screening). For the 26 patients for whom PDL-1 positivity assessment was available (cut-off ≥1%) OS did not change when compared to the overall unselected population (5.22 months in the Nivolumab group) and the activity of immunotherapy in PDL-1 negative patients was fully superimposable with an OS of 6.05 months in the Nivolumab arm. At the moment no further data are available about other biomarkers (i.e., MSI status) but it is absolutely noteworthy that this is the first positive randomized phase 3 study of an immune checkpoint inhibitor in patients with advanced gastric cancer.

Anyhow ATTRACTION-2 is a fully Asian trial and a possible debate could arise about whether its data could be transferred or not to the western population. GC patients from Asia and from western countries are known to have different clinical outcomes [85,86], these differences have been conventionally attributed to different clinical management and disease stage. Instead, recent evidences suggest that gene expression differences exist among Asian and non-Asian gastric adenocarcinomas and different molecular signatures influence clinical outcome, especially concerning tumor immunity [87].

However, we should consider that Pembrolizumab has been successfully used also in the western population (KEYNOTE-059) and even Nivolumab has been tested in a non-Asian population. As a matter of fact in the gastric cohort of phase 1/2 trial CheckMate-032 [88] a favorable and promising activity of Nivolumab +/− Ipilimumab (a fully human monoclonal IgG1-k against CTL antigen 4) has been described in a fully Western population (160 heavily pretreated patients). Even in the Nivolumab single agent arm (quite similar to Attraction-2 experimental arm), ORR was 12% in the unselected population and 19% in PDL-1 positive patients, although outcomes have been worse than in the combination arms, and a survival rate of 39% has been observed at one year.

Tan et al. [89] published a large retrospective investigation of more than 1600 gastric cancers from six Asian and three non-Asian cohorts demonstrating that tumor immunity signatures differ significantly between Asian and non-Asian cancers. These different tumor immunity signatures, especially related to T cell function, might explain the geographical differences in clinical outcome always observed and might condition different responses to immunotherapy.

In particular Non-Asian gastric cancer seemed to be associated with enrichment of tumor infiltrating T-cells as well as T-cell gene expression signatures, including CTLA-4 signaling, while Asian gastric cancers had a significantly higher numbers of cells positive for neutrophil markers. 

Asian and Caucasian GCs had distinct immune-related components, intratumoral immune, and inflammation cells populations.

Investigators also analyzed whether these differences might be due to different EBV and MRR status, both conditions notoriously related to high load of infiltrating lymphocytes [90], but no significantly differences have been demonstrated between the two cohorts.

Due to the retrospective nature of this study, results need to be further validated but we should consider that western population seems to have an “immune-environment” more favorable to immunotherapy and a stronger immune-signature, even if, as already reported, the biggest immune-oncology phase 3 trial currently available (ATTRACTION-2) has been conducted only on a entirely Asian population.

Results of ongoing immune-oncology trials [91,92] on western populations are hopefully awaited, but the design of future gastric cancer trials should consider an accurate patient selection and tumor immunity differences in patients from different geographic regions.

## 7. Conclusions

Clinical application of new advances and recent molecular classifications is still disappointing. Tumor heterogeneity and the still imperfect understanding of the complex tumor biology represent a brake to the definitive overcoming of the “one size fit-all” era also for gastric cancer.

With the exception of first accelerated anti-PD-1 drugs approval, at current time, as mentioned above, only two target therapies have been approved and are currently used as a standard of care: Trastuzumab and Ramucirumab.

When compared to the big amount of molecular drivers identified and tested for GC, these advances represent still a small step to that more contemporary and comprehensive clinical approach, called “precision medicine”, to which genomic heterogeneity is a stubborn barrier.

New treatments are urgently needed in order to give patients more benefits from new drugs and new technologies available. What molecular classifications can already teach us is that in the next future it will not be possible to give patients a same treatment in an unselective manner and that a more rigorous selection of patients will be mandatory.

For example evidence clearly identified two subgroups of GC, as characterized by MSI-H and EBV-positive status, which may benefit from immunotherapy and for whom front-line anti-PD1 drugs could be pursued, in addition to other hallmarks (PD1 status, TMB) that will drive therapeutic approaches and future research.

On the other side it is also possible to identify another subset of GCs (GS, MMT/EMT) poorly responsive to immunotherapy and in which for example the development of inhibitors of cMET pathway, Rho-kinase, PI3K/mTOR pathway should be encouraged as demonstrated by the anti-claudin line of research, which seems particularly promising in the selected population.

Another challenge is represented by the development of predictive biomarkers of response also using the ctDNA technology, a procedure that will need to be validated and studied in deep but that could help identifying patients with a high risk for progressive disease early and their possible resistance mechanisms.

The analysis of ctDNA might also help detecting genomic alterations not detected in primary tumors at baseline, considered the evidence of a possible significant discrepancy within a same primary tumor and between primary tumors and metastatic sites genomic alterations, a discrepancy potentially determining the targeted therapies failure [93].

As already tested in other cancer types [94], also the use of patient-derived xenograft (PDX) models could help in the search of these predictors. Analyzing non-responder PDXs, for example, we could identify those mechanisms that determine resistance to the tested therapy facilitating the ideation of dedicated prospective trials. As a matter of fact gastric PDX platforms are already available and first studies have been conducted [55,95].

A complementary interesting possibility could be represented by the use of “tumor organoids” [96] utilized as an alternative model for the research in order to verify ex vivo the sensitivity of tumor cells to targeted therapies. Furthermore PDXs and organoids can help in understanding why in different tumor contexts effective targeted agents are poorly active in different cancer types (i.e., anti-HER2 therapy in colorectal and gastric cancer).

GC is a collection of different molecular entities and its landscape is enormously complex, but recent efforts and knowledge laid the foundations for the development of more modern and solid therapeutic approaches and clinical trials, also taking advantage of new preclinical models to revalue promising drugs that failed in the past years.

Molecular classifications, especially TGCA and ACRG, opened the doors wide on the complete comprehension of the complex genetic landscape of gastric cancer and on the way to the full application of precision medicine also for this malignancy, even if at current time they still appear as separated and isolated systems, while a standardization and a “common strategy” approach would be desirable.

The biggest challenge for the next future will be to understand which cancer subgroup deserves a specific targeted agent and to design clinical trials tailored on these subgroups, in order to transfer the molecular classifications acquisitions into the clinical practice and to minimize the numbers of patients who receive a systemic treatment without any molecular selection.

## Figures and Tables

**Table 1:** Major target-oriented phase II/III trials in gastric and esophagogastric adenocarcinomas.

Trial	Phase	Setting	Target	Arms	*N* Patients	Primary Endpoint	Result
**ToGA**	III	1st line	HER2+	CF/CX ± Trastuzumab	594	OS	Positive
**JACOB**	III	1st line	HER2+	CF/CX+ Trastuzumab ± Pertuzumab	780	OS	Negative
**GATSBY**	II/III	2nd line	HER2+	Taxanes ± TDM-1	345	OS	Negative
**LOGIC**	III	1st line	HER2+	CapeOX ± Lapatinib	545	OS	Negative
**TyTAN**	III	2nd line	HER2+	Paclitaxel ± Lapatinib	261	OS	Negative
**EXPAND**	III	1st line	EGFR (unselected)	CX ± Cetuximab	894	PFS	Negative
**REAL-3**	III	1st line	EGFR (unselected)	EOC ± Panitumumab	553	OS	Negative
**METGastric**	III	1st line	MET+	Folfox ± Onartuzumab	562	OS	Negative
**RILOMET-1**	III	1st line	MET+	ECX ± Rilotumumab	609	OS	Negative
**SHINE**	II	2nd line	FGFGR2+	Paclitaxel ± AZD4546	71	PFS	Negative
**FAST**	IIb	1st line	CLDN18.2+	EOX ± Claudiximab	161	PFS	Positive
**AVAGAST**	III	1st line	VEGF	CX ± Bevacizumab	774	OS	Negative
**AVATAR**	III	1st line	VEGF	CX ± Bevacizumab	202	OS	Negative
**REGARD**	III	2nd line	VEGFR2	Ramucirumab vs. Placebo	355	OS	Positive
**RAINBOW**	III	2nd line	VEGFR2	Paclitaxel ± Ramucirumab	665	OS	Positive
